# Managing Relevant Clinical Conditions of Hemophilia A/B Patients

**DOI:** 10.3390/hematolrep15020039

**Published:** 2023-06-07

**Authors:** Massimo Morfini, Jacopo Agnelli Giacchiello, Erminia Baldacci, Christian Carulli, Giancarlo Castaman, Anna Chiara Giuffrida, Giuseppe Malcangi, Angiola Rocino, Sergio Siragusa, Ezio Zanon

**Affiliations:** 1 Italian Association of Haemophilia Centers (AICE), 21121 Milan, Italy; 2 Hemostasis and Thrombosis Center, Azienda Ospedaliera SS Antonio e Biagio e Cesare Arrigo, 15121 Alessandria, Italy; 3 Haematology, “Umberto I” Policlinico, Department of Translational and Precision Medicine, Sapienza University of Rome, 00118 Rome, Italy; 4 Department of Orthopaedic Surgery, Orthopaedic Clinic, University of Florence, 50121 Florence, Italy; 5 Department of Oncology, Center for Bleeding Disorders and Coagulation, Careggi University Hospital, 50121 Florence, Italy; 6 Transfusion Medicine Department, AOUI Verona, 37100 Verona, Italy; 7 UOSD Centro Emofilia e Trombosi, Azienda Ospedaliero Universitaria Policlinico di Bari, 70121 Bari, Italy; 8 Haemophilia and Thrombosis Centre, Haematology, S.M. di Loreto Nuovo Hospital, 80121 Naples, Italy; 9 Department PROMISE, University of Palermo, 90121 Palermo, Italy; 10 Haemophilia Centre, General Medicine, Padua University Hospital, 35121 Padua, Italy

**Keywords:** hemophilia A, hemophilia B, rFVIII SHL&EHL, surgery, FVIII inhibitors, bypassing agents, prophylaxis, genetic modifiers

## Abstract

The Medical Directors of nine Italian Hemophilia Centers reviewed and discussed the key issues concerning the replacement therapy of hemophilia patients during a one-day consensus conference held in Rome one year ago. Particular attention was paid to the replacement therapy needed for surgery using continuous infusion (CI) versus bolus injection (BI) of standard and extended half-life Factor VIII (FVIII) concentrates in severe hemophilia A patients. Among the side effects, the risk of development of neutralizing antibodies (inhibitors) and thromboembolic complications was addressed. The specific needs of mild hemophilia A patients were described, as well as the usage of bypassing agents to treat patients with high-responding inhibitors. Young hemophilia A patients may take significant advantages from primary prophylaxis three times or twice weekly, even with standard half-life (SHL) rFVIII concentrates. Patients affected by severe hemophilia B probably have a less severe clinical phenotype than severe hemophilia A patients, and in about 30% of cases may undergo weekly prophylaxis with an rFIX SHL concentrate. The prevalence of missense mutations in 55% of severe hemophilia B patients allows the synthesis of a partially changed FIX molecule that can play some hemostatic role at the level of endothelial cells or the subendothelial matrix. The flow back of infused rFIX from the extravascular to the plasma compartment allows a very long half-life of about 30 h in some hemophilia B patients. Once weekly, prophylaxis can assure a superior quality of life in a large severe or moderate hemophilia B population. According to the Italian registry of surgery, hemophilia B patients undergo joint replacement by arthroplasty less frequently than hemophilia A patients. Finally, the relationships between FVIII/IX genotypes and the pharmacokinetics of clotting factor concentrates have been investigated.

## 1. Introduction

In recent years, because of the large availability of new replacement therapies for hemophilia A and B based on SHL or EHL rFVIII and rFIX concentrates in developed countries, clinicians and patients have had new opportunities to tailor treatments according to patients’ lifestyles, bleeding patterns, and clinical conditions. These situations are often managed according to the clinician’s expertise, sometimes lacking clear scientific evidence. There are still circumstances in which clinicians need to attain more scientific evidence, which requires a peer-to-peer discussion. Moreover, even if a body of solid data is available, the local healthcare conditions of Hemophilia Centers may represent a challenge for adhering to national and international guidelines. A panel of ten Italian expert treaters of hemophilia addressed the following specific issues: 1—the prevention of bleeding during surgery; 2—the prophylaxis of spontaneously occurring bleedings; 3—the relationships between the genotypes and phenotypes of hemophilia A and B. Each participant in the meeting presented his opinion according to his expertise related to each issue. This text is the result of a general discussion.

## 2. Replacement Therapy with FVIII/IX Concentrates on People with Hemophilia A/B Undergoing Surgery

The establishment of a multidisciplinary team (MDT) should be the first aim to be achieved. This is also explicitly emphasized by the current WFH guidelines [1]. The MDT should coordinate the activities of the professional workers before, during, and after surgery, namely: (a) the assessment at least of the individual baseline in vivo recovery (IVR) and the trough of the FVIII or FIX level during the postoperation period; (b) the pre- or postoperation information provided to the patient and family members; (c) the surveillance on the development of neutralizing inhibitors, the prevention of thromboembolic events, and other side effects. As outlined by the WFH guidelines, it is also necessary to improve the treatment of hemophilic patients by creating a well-trained MDT. It is very important to have a surgeon and an anesthesiologist both with specific experience in hemophilia management. A well-detailed protocol for any surgery should be planned in advance to prevent some problems that may arise, such as intra- or postoperative bleeding or thromboembolic complications.

### 2.1. Continuous Infusion or Bolus Infusion to Provide Perioperative Replacement Therapy

Clinicians agree that perioperative prophylaxis by continuous infusion (CI) has some advantages, such as constant and safe FVIII/FIX levels, a lower risk of bleeding, and the elimination of FVIII/IX spikes, reducing the thrombotic risk and the costs of surgery. CI can allow a decrease of about 30–36% of the total amount of FVIII needed for surgery [2], especially in children [3]. The sparing effect of CI is particularly evident during the surgical prophylaxis of hemophilia A patients with inhibitors, treated with rFVIIa infusion: the tall peaks of FVIIa of bolus infusions (BI) can be eliminated by CI [4]. However, some disadvantages of the CI of rFVIIa cannot be underestimated. Currently, the major issues in this setting are: (a) CI is an off-label procedure; (b) the risk of bacterial contamination and possible degradation of rFVIIa during the CI; (c) phlebitis at the site of the infusion venous access and/or secondary thromboembolic events. In addition, there are different opinions among the treaters about the risk of developing FVIII inhibitors using CI [5,6,7], since the use of high doses of FVIII has been suggested to be a risk factor for inhibitor development [8,9]. CI requires the hematologist’s supervision and laboratory monitoring, with daily control of the FVIII plasma level. Based on existing meta-analyses, there is no evidence that CI is better than BI, and in both cases, it is possible to identify some advantages and disadvantages (Table 1). The nurses should be trained in the management of pumps for CI; furthermore, it should also be considered that these devices can restrict the mobility of the patient after the operation when a portable mini pump is not available. Some physicians emphasize the need to use concomitant administration of 10 mL/h saline solution by the same line of the CI and Doppler examination to rule out the occurrence of venous thrombosis. These factors significantly limit the number of Hemophilia Centers with enough resources and staff to implement this strategy of administration of replacement therapy. In the general opinion, BI is to be preferred, especially in some small Hemophilia Centers, because it is the standard allowed type of administration, and the cost and the management of the infusion pumps may be a concern. BI allows health and nursing staff to easily manage the patients in the postoperative phase, while the patient has more freedom of movement after the operation, not being connected to an infusion pump. The physicians should evaluate whether they would prefer bolus or continuous infusion, should adequate resources be present. From the sharing of experiences, it emerges that the major obstacle to the implementation of CI is the lack of pieces of evidence: it is an off-label treatment. In any case, there is a significant amount of literature that provides support for the use of CI [2,6,10,11,12]. The CI of moroctocog alpha has been shown to provide safe hemostasis, with lower factor consumption and an excellent postoperative outcome in 92% of patients as assessed during the first postsurgery week [13].

### 2.2. Tailoring the Replacement Therapy during Surgery in People with Hemophilia A

The possibility of having constant levels of circulating FVIII/IX during and after surgery may limit the bleeds caused by their fluctuations and decrease the amount of replacement therapy. The assessment of the individual pharmacokinetics of the FVIII/IX concentrate during the preoperative period may provide an accurate evaluation of the PK parameters, such as the clearance and half-life of the FVIII/IX product. These parameters will be very useful to maintain a safe plasma level during the peri- and postoperative periods. The WFH guidelines suggest having an adequate supply of the concentrate according to pharmacokinetic parameters previously determined before starting surgery to be able to treat any accidental bleeds [1]. Indeed, a review of the literature suggests that the risk of developing inhibitors after a switch to another product is limited [14], although surgery is a facilitating condition. However, Iorio’s study [15] looked at previously treated patients (PTPs) who developed inhibitors and reported that 22% had undergone surgery in the previous two months. In any case, the infusion of different concentrates should be avoided during the postoperative period unless clinical conditions are worsening because of severe bleeds or other emergencies. Finally, there is a need for an accurate standardization of the treatment of patients who will undergo surgery, to keep an adequate plasma level of FVIII and avoid the risk of bleeding events. The increase in the average age of the hemophilic population leads to an increase in comorbidities and related concomitant medications, which may affect the pre- and postoperative conditions. The stress of surgery itself, the tissue injuries, and blood clotting at the site of the operating field may trigger systemic or local activation of fibrinolysis. The generated plasmin may decrease the clotting factor plasma concentration and impair the effectiveness of the replacement therapy. For these reasons, antifibrinolytic treatment with tranexamic acid (1 g every 8 h daily) has been frequently added to replacement therapy during the pre- and postsurgery periods. The risk of venous thrombosis in people with hemophilia undergoing surgery is very limited, but the association of tranexamic acid may be an important cofactor. Some people with hemophilia may have a particular risk factor for venous thromboembolic events, thus requiring antithrombotic prophylaxis. Patients should move lower limbs as soon as possible and/or pneumatic compression should be performed. Antithrombotic prophylaxis with LMW heparin can be done with great attention to avoid the risk of secondary bleeding.

### 2.3. Tailoring the Replacement Therapy during Surgery in People with Hemophilia B

As far as the treatment in people with hemophilia B undergoing surgery, the FIX concentrate most utilized for over 30 years, with excellent results, is nonacog alfa. About 40% of the loading presurgery dose of FIX flows in the extravascular space and afterward back into the plasma compartment [16,17]. This is the reason why the nonacog alpha shows a more biphasic decay curve and why its half-life is longer. This characteristic can allow a long interval (12–24 h) between postsurgery boluses. As far as the dosing is concerned, the interval between the boluses, after the same loading dose, depends on the planned trough of FIX:C to be maintained during the postoperative period. Similarly, the intervals between rFIX EHL infusions should be shorter or longer according to the efficacy to maintain hemostasis as clinically assessed. In both cases, the higher cost of rFIX EHL concentrates may increase the cost/effectiveness of replacement therapy [18]. We should consider that about 40% of the administered nonacog alpha flows in the extravascular space, where it is in equilibrium with FIX:Ag [19]. According to the two-compartment model, the concentrate can flow back from the extravascular into the plasma compartment (the so-called “retrograde clearance”), allowing a longer half-life [20,21,22]. This provides a slower decay curve with a long beta half-life and a prolonged safe trough. At the steady state, the bolus treatment by an rFIX SHL can allow a plasma concentration such as that provided by the continuous infusion. It is agreed that the principal goal is to acquire optimal values that avoid postoperative bleeding. The different surgical protocols, with or without a tourniquet, or simultaneous bilateral surgeries, may influence the bleeding and the amount of needed replacement therapy.

### 2.4. Standardized Protocol for the Replacement Therapy during Surgery

It is important to standardize procedures to better measure the potential benefit of one drug over another. One suggestion is to perform a detailed analysis of the various surgeries, concerning the need for FVIII or FIX. This issue is important because the population of people with hemophilia who undergo surgery is now quite large. Regarding the long-acting treatments, the rFIX EHL concentrates may allow for keeping the FIX level around 50% during the first days following surgery. It emerges that these drugs have determined a significant reduction of rFIX concentrate consumption during orthopedic surgery; however, with a three-fold increase in the cost of treatment [18]. There is no homogeneity of thought about the cost/benefit ratio of the replacement therapy for hemophilia: some physicians argue they should first evaluate the effectiveness of the drug and the achievement of the therapeutic goal rather than the cost. According to the opinion of other treaters, great attention must be paid to the cost/efficacy ratio of the treatment.

### 2.5. Anticoagulation Prophylaxis in Hemophilia Patients

The risk of bleeds is the major concern during surgery in severe hemophilia patients. Of course, supranormal FVIII/IX postinfusion levels must be avoided: this risk must be considered when bolus instead of continuous infusion has been implemented. Intermittent pneumatic compression devices and early mobilization of the patient should be considered in the postsurgery period of hemophilia patients. Low-molecular-weight heparin can be considered after orthopedic surgery as total knee or ankle arthroplasty.

After the implementation of prophylaxis and the increase in life expectancy, hemophilia patients may also have cardiovascular diseases. Mild and severe hemophilia A patients on prophylaxis should undergo anticoagulant therapy according to their baseline FVIII/IX and stroke risk [23,24]. Elderly hemophiliacs can undergo diagnostic invasive procedures and drugs provided that tailored prophylaxis has been implemented [25,26]. The need of correcting the hemostatic defect using adequate replacement therapy before any anticoagulant therapy and/or invasive procedures is strongly recommended in acute coronary syndrome management [27].

## 3. Management of People with Moderate and Mild Hemophilia A

The use of desmopressin (DDAVP) may successfully achieve the prophylaxis of bleeding in moderate or mild hemophilia A. This drug releases the FVIII/VWF complex stored in the Weibel–Palade bodies of the endothelial cells. An FVIII plasma level of around 40–60 IU/dL can be achieved after about 0.5–1 h from subcutaneous or intravenous administration of the drug (0.3 µg/kg). The drug can also be self-administered by the patient using the nasal spray formulation as a prophylactic treatment after minor surgery and to attend sports and other demanding physical activities. DDAVP is very cheap compared to the costs of FVIII/VWF concentrates. The treatment must be started 1–2 h before the elective surgery, and it is not suitable for emergency treatment when a rapid increase of the FVIII/VWF complex is mandatory. The drug can also release the plasminogen activator from the endothelial storage. The contemporary oral administration of tranexamic acid (1 g × 3 times, daily) is useful in counteracting secondary hyperfibrinolysis. Because the half-life of the elicited FVIII/VWF complex is short, lasting around 8–12 h [28], the DDAVP must be administered every 12–24 h. However, the FVIII/VWF endothelial stores are exhausted after a few days, but the treatment can be started again after 1–2 days of interruption. In conclusion, DDAVP treatment is recommended for minor surgery in people with moderate and mild hemophilia A who respond to its treatment limited to a postoperative period not longer than 6–7 days. In case of breakthrough bleeding, the patients must be switched to replacement therapy with FVIII concentrates. DDAVP, reducing the exposure to exogenous FVIII, may decrease the risk of inhibitors, first in patients affected by some specific mutations causing moderate or mild hemophilia, then in those at high risk of the development of FVIII inhibitors. The risk of water retention by DDAVP and consequent hyponatremia must be carefully considered in the prolonged treatment.

## 4. Management of Bleeds of People with Hemophilia A and FVIII Inhibitors: The Role of Bypassing Agents

Activated prothrombin complex concentrate (APCC) and activated recombinant FVII (rFVIIa) have been developed to allow thrombin generation and the promotion of blood coagulation, notwithstanding the inhibition of FVIII by specific antibodies, even at a high titer [29]. The activation of FX to FXa is achieved by intrinsic tenase (a complex containing FX, the activated FIXa, and its cofactor FVIIIa) or by extrinsic tenase (tissue factor linked to FVIIa). Both APCC and rFVIIa have been used with success in the prevention of bleeding, as reported in several clinical trials. The high annualized bleeding rate (ABR) of 28.7 observed in people with hemophilia A treated on-demand significantly decreased to 7.9 during prophylaxis (85 ± 15 IU/kg every other day) with APCC [30]. Twenty-two HA patients with FVIII inhibitor were randomly allocated to daily treatment with two different rFVIIa doses, one daily megadose of 270 µg/kg vs. two to three infusions of 90 µg/kg daily, for three months. The frequency of bleeding decreased, respectively, by 45% and 59% compared with the previous conventional on-demand treatment (*p* < 0.0001), but without difference between the two doses [31]. According to the outcomes of a retrospective study conducted on 86 patients from 14 countries, the decrease in bleeding after starting prophylaxis with rFVIIa was 46–52% [32]. The rFVIIa and APCC are very helpful for the treatment of spontaneous and surgical bleeding in hemophilia patients with inhibitors, thus making surgery possible. The CI of rFVIIa showed high efficacy and safety in the prevention of peri- and postoperative bleeding during total arthroplasty performed in people with hemophilia A and the FVIII inhibitor [33,34]. The rFVIIa is also very useful in nonorthopedic surgery [35].

A FEIBA versus Novoseven Comparative (FENOC) crossover study has been conducted in 48 hemophilia A patients with FVIII inhibitors to compare the clinical outcome and cost of the activated prothrombin concentrate (aPCC) vs. rFVIIa in the stopping the joint bleeds. According to sensitivity analysis, the data showed that patients reported a nonstatistically significant higher efficacy of aPCC, due to the large interindividual variation. Both treatments determined a similar reduction of pain up to 48 h. The cost of treatments with rFVIIa were higher than those of aPCC [36].

## 5. Prophylaxis in People with Severe Hemophilia A

Hemophilic arthropathy can be started even after the first hemarthrosis, [37] which represents the start of recurrent bleedings, in a vicious circle finally ending in joint ankylosis. The first hemarthrosis occurs in 75–90% of severe hemophilia A patients within the first 2–3 years of life. The knees and ankles, bearing the body weight, are the more frequently affected joints. For these reasons, prophylaxis should be started as soon as possible (primary prophylaxis), at least after the first hemarthrosis in all people affected by severe hemophilia A. The occurrence of joint damage in severe (FVIII < 1 IU/dL) but clinically mild patients, or vice versa, underlines the crucial role of the careful clinical observation of hemophiliac children by their parents and doctors! Up to now, in Italy, early prophylaxis is the first choice of treatment in about 70% of people with hemophilia A [38]. The large availability of virus-inactivated serum and albumin-free second-generation recombinant concentrates, or of the third-generation free-of-animal raw materials, improved the confidence of the treaters in prophylaxis. Among them, particular attention was given to moroctocog alpha because it was the first recombinant B-domain-deleted concentrate [39]. This modification of the transduced molecule allows increased production of FVIII by CHO cells, with a more effective transfection process. This is the reason why most fourth generation rFVIII EHL concentrates, except rurioctocog alfa pegol, are all B-domain-deleted. After some concerns about the immunogenicity of this modified FVIII molecule were raised [40], different clinical observations of the pros and cons of this issue have been reported [41,42]. Most physicians worldwide seem not to have been worried about the risk of inhibitor development by the usage of moroctocog alfa [43], since the risk is very similar to that of other rFVIII concentrates [44]. Even though secondary prophylaxis is less effective than primary prophylaxis in preventing the development of chronic arthropathy, it has been implemented in adolescent or adult people with hemophilia who did not undergo primary prophylaxis and then developed target joints. Patients who are practicing sports are strongly recommended to undergo prophylaxis instead of on-demand treatment. Prefilled syringes with FVIII concentrate can help facilitate the injections for patients attending training or sports competitions.

### 5.1. Tailoring Prophylaxis in People with Moderate Hemophilia A or Sedentary People with Severe Hemophilia A by rFVIII SHL Concentrates

The interpatient variability of rFVIII pharmacokinetics is very large. The half-life of moroctocog alpha resulted in 17.69 ± 13.27 h using the one-stage assay and the moroctocog laboratory standard [45]. About 30% of people with severe hemophilia A showed longer-than-expected FVIII half-life because the distribution and elimination phases of the infused concentrate do not depend only on the baseline FVIII level, but also on the VWF plasma level and the different polymorphisms of other genes [46,47,48,49,50]. These patients can undergo twice- instead of thrice-weekly prophylaxis. However, accurate individual evaluation of the PK of the FVIII concentrate is advised to better tailor prophylaxis. One size and one product do not fit all! The individualization of the prophylactic regimen may allow them to personalize the treatment and spare the dosing of the concentrate. The preference of patients is for a lower frequency of administrations. Unfortunately, the small increase of the HL (about 2–3 h) of the new rFVIII EHL concentrates may allow an interval between the infusions generally not longer than 5 days in the large majority of patients. Due to the well-known large interpatient variability of the FVIII HL, the lower frequency (one infusion every 10 days) is limited to a few patients. A study performed in an Iranian cohort [51] showed that in some patients it was required to increase the standard dosage of weekly prophylaxis according to the rate of bleeding events. However, we are far from the standardization of treatment, and the same dose does not fit all patients’ needs.

Within the treatments with rFVIII EHL, good outcomes were obtained with pegylated recombinant factors: in pediatric patients, rurioctocog alfa pegol allowed the frequency of infusions to be reduced to two days a week [52], and sometimes using damoctocog alfa pegol at a dose of 50 ± 10 IU/kg once every 5 days reduced the frequency of hemarthrosis [53]. Since the introduction of the new FVIII monoclonal purified or recombinant concentrates, great attention has been focused on the pharmacokinetic profile [54,55]. The PK parameters do not seem to be influenced by the F8 genotype of the recipients. There is great variability among the treated patients, even if it seems to be quite reproducible in the same patient (personal data). The results of the comparative PK between the rFVIII SHL and plasma-derived FVIII showed average bioequivalence [45]. However, the pharmacokinetic results showed, in some patients, a higher-than-expected mean residence time of the rFVIII concentrates [56]. The most important PK parameter when setting up the treatment by repeated infusions is the total body clearance, i.e., the plasma volume made free of the factor infused per unit of time. Knowledge of the clearance improves the doctor’s perspective, so that it is no longer based only on the time elapsed between bolus administrations, but also on the trough to be maintained according to the patient’s characteristics: lifestyle, physical activity, and personal needs because of familial and work commitments. Finally, attention has been drawn to factor consumption when using SHL or EHL drugs. Several studies show that there is a reduction in factor consumption during surgery with EHL products [57,58,59,60]. The choice of the best product is based only on patient variability, and currently there is insufficient evidence about the differences among the products because of the lack of good quality comparative studies. In addition, the limited available data were attained from heterogeneous populations. In this area, the perception is that clinical practice will provide more in the long-term reliable information. Another element that should be emphasized is that the use of prophylaxis should be concomitant with a frequent clinical re-evaluation of the response, using reliable parameters. So far, physicians have used the ABR as a parameter. The goal of prophylaxis is to attain an ABR closer to zero in the larger population of patients, together with a more reduced frequency of FVIII infusions: the best ratio of cost/effectiveness. Unfortunately, the implementation of rFVIII EHL drugs in routine clinical practice has shifted the focus to the increased cost of therapy despite the clinical outcome [61]. There is a need to make the regulatory authorities fully aware of the new treatments for the different types of hemophilic patients, regardless of the cost. As far as the therapeutic aim of the ABR value equal to zero is concerned, there is an open debate. Some physicians argue that, regardless of the drug used, the ABR value equal to zero implies an increase in quality of life, and reduces the risk of developing arthropathy and the consequent need for surgery. It should therefore be a driving factor in clinical practice. Other physicians, however, raise some concerns: there are cases (especially in pediatric age) in which, despite an ABR equal to zero, joint damage may still occur.

### 5.2. The Issue of joint Microbleeds during Prophylaxis

The MJ Manco-Johnson study on the effect of prophylaxis to prevent hemophilic arthropathy [62] represents a milestone in the treatment of hemophilia A patients. Sixty-five hemophilic children A, mean age 1.6 years, were enrolled and followed until the age of 6 years. Patients with a minimal number of previous joint hemorrhages (0–5) were randomly assigned to prophylaxis (n = 32, 25 IU/kg every other day) or to on-demand high-dose treatment (n = 33, total 80 IU/kg in three doses). Changes in bone or cartilage structure of the ankles, knees, and elbows examined by radiography or MRI were considered the primary outcome. At the end of the study, 93% of patients on prophylaxis in contrast to 55% of those on on-demand therapies showed no change in joint structure on the MRI (*p* = 0.006). The total ABR was 3.7 ± 6.24 among patients on prophylaxis and 17.69 ± 9.25 in on-demand treatment patients, while the joint ABR was 0.63 ± 1.35 and 4.89 ± 3.57, respectively. A weak correlation between hemarthroses and the MRI outcomes has been observed in a few patients who did not report hemarthroses but had high MRI scores, probably because of subclinical bleeds. These observations suggest the need for accurate and early prophylaxis in severe hemophilia A patients, independently of the clinical evidence of bleeds. On the contrary, few patients reported more than 10 hemarthroses without bone or cartilage changes on the MRI. Joint changes presumably induced by subclinical joint bleedings have been observed in patients on prophylaxis [63], or with no severe hemophilia [64] or with mild arthropathy [65]. Recently, hemosiderin deposits have been reported using an MRI in 7 of 43 (16%) joints in patients on lifelong prophylaxis and who were clinically asymptomatic [66]. There is, therefore, an absolute need to identify an objective parameter of measurement on micro bleedings. The issue may become more crucial with gene therapy, in which the treatment is given only once and does not exclude continuous monitoring just to control subclinical bleeds. In summary, physicians agree that the primary goal is zero ABR, especially in the younger population. When the new therapies that achieve FVIII levels above 20% for a long time enter normal clinical practice, the goal will be to control microbleeds to better avoid joint complications. At present, however, the primary need is to identify the plasma levels of FVIII, which ensure no bleeds occur at all. It will also be important to better understand the etiopathology of synovitis, even during prophylaxis with rFVIII EHL concentrates or gene therapy.

### 5.3. Shared Decision-Making to Treat People with Hemophilia B besides Prophylaxis

People with moderate hemophilia B can avoid being treated frequently if their lifestyle is not excessively active, but prophylaxis must be started as soon as the first joint bleeding occurs. Few people with severe hemophilia B are treated on-demand because the treaters start prophylaxis as soon as possible. Because of the good half-life of rFIX SHL concentrates in about 30% of severe hemophilia B patients, even a once-weekly infusion regimen may be enough to reduce the frequency of bleeding [67]. Sometimes, if patients are not capable of self-treatment or live far from the hospital, on-demand therapy is the first choice. Single-dose pharmacokinetics is a valid tool to evaluate the individual response to replacement therapy. AUC, half-life, and clearance can provide very useful information about the tailoring of the treatment, but lifestyle is the principal parameter to tailor the individual prophylactic regimen of each patient. The bleeding phenotype and the logistical difficulties related to the age of the patient, the venous access, the family’s collaboration, and the constant supply of the concentrate are the major issues to be considered before starting prophylaxis in young children or adolescents. Some older people with severe hemophilia B previously treated on demand, who in the past developed arthropathy, frequently prefer to avoid the cumbersome weekly prophylaxis, notwithstanding the advice of their physician.

## 6. Genetic Modifiers the Clinical Characteristics of Hemophilia A/B and Pharmacokinetics of FVIII/IX Concentrates

### 6.1. Hemophilia A

The large interpatient variability of PK outcomes of all FVIII/IX concentrates observed even in well-controlled PK designs may depend on the different genetic characteristics of each person with hemophilia. The derangement of the F8 gene by inversions of intron 22 or 1, occurring in about 50% of people with hemophilia A, results in less than 1 IU/dL FVIII baseline concentration, making these patients more disposed to bleeding. As far as the PK outcomes of the FVIII concentrates, patients with or without intron 22 inversion did not show significant differences (personal data). Apart from the FVIII genotype, other genetic polymorphisms seem to be able to determine the pharmacokinetics of infused FVIII concentrates. Hemophilia patients with blood group 0 showed a faster decay of the infused FVIII due to the faster clearance of endogenous VWF through anti-A and B agglutinins [68]. LDLR c.1773C/T polymorphisms were associated with different constant rates of movement of the infused FVIII from the plasma to the extravascular compartment (K 1–2), and vice versa (K 2–1), and the alpha distribution phase. FVIII clearance and the volume of distribution at steady state were instead associated with the LDLR c.81C/T polymorphism [47]. ASGR2 c.-95TT homozygotes showed long alpha HL (3.60 h) and the c.-95TC heterozygotes showed about a 25% shorter MRT (18.5 h) and a 32% shorter Beta HL (13.5 h). The ASGR2 genotype influence was statistically significant, independently from the ABO genotypes and the von Willebrand factor (VWF) antigen levels. They were responsible for a 14% variability of the MRT, 15–18% of the beta HL, and 22% of the alpha HL [48]. The CLEC4M (C-type lectin domain family 4 member) genotype is responsible for the binding and internalization of the infused rFVIII concentrates. According to the two-compartment model PK of the plasma-derived and full-length rFVIII concentrates, CLEC4M rs868875A/G genotype groups showed different results. Among genotypes, AA, AG, and GG, the elimination rate constant K 1–0 results were significantly different (*p* < 0.001), as were the K 1–0 HL and the beta elimination rate constant. The CLEC4M G-carriers/blood group 0 genotypes showed faster FVIII clearance (mean 7.1 ± 2.2 mL/h/kg SE) than in the G-carriers/non-O (mean 2.4 ± 0.3 mL/h/kg SE) (*p* = 0.038) [69].

### 6.2. Hemophilia B

As far as hemophilia B is concerned, missense mutations are prevalent (55%). In contrast to what is observed in patients with hemophilia A, the complete deletions of the F9 gene are only 17% [70]. This is the reason why hemophilia B is generally considered less severe than HA [71,72,73]. Nevertheless, different opinions have been raised on this issue [74,75]. On the other end, arthropathy and the need for total joint replacement seem to be less frequent in hemophilia B than in hemophilia A [76,77]. Different outcomes were raised from a very large Taiwan database (782 HA and 153 HB patients) [78]. The relationship between different F9 genotypes and the PK of nonacog alfa [20] has been recently extensively investigated (GePKHIS protocol; Eudract ID2017-003902-42) [79]. Recurrent substitutions at FIX activation sites (R191–R226) are associated with variable FIX:C and FIX:Ag levels. The alpha and beta half-lives of nonacog alpha are correlated with the FIX:Ag level, produced in vitro by the patient’s mutation R191/r226. The MRT was quite long (79.4 h, range 44.3–114.5 h) in hemophilia B patients with the r191/r226 substitutions [79]. Theoretically, more detailed information about the genetic characteristics of each patient might allow a genetically driven replacement therapy for hemophilia A and B.

## 7. The Effects of the Different Tissue Distribution of FVIII and FIX on Replacement Therapy

About 40% of the FIX circulating in the body is located in the extravascular space in equilibrium with the FIX of the plasma space [17]. The low molecular weight of FIX (57 Kd) allows the movement of the molecule from the plasma compartment to the extravascular space. This is the reason why the infused FIX has a low in vivo recovery (IVR), around 1.0 IU/dL per IU/kg infused, and a fast clearance. The flow back from the extravascular space (retrograde clearance or CLD2) maintains a prolonged permanence of infused FIX in the plasma compartment, as shown by the long beta HL (32.9–53.5 h) and the MRT (42.9–64.0 h) observed in the recent PK study of nonacog alpha [79]. Furthermore, because of the presence of FIX in the vessels’ epithelial cells and subendothelial structures [20,80], people affected by hemophilia B may be less susceptible to bleeding and hemarthrosis compared to hemophilia A subjects [68,69,70,71,72,73]. On the contrary, FVIII is a very large molecule (330 Kd), and about 95% of infused FVIII is soon bound to circulating VWF as well as 5% to subendotelial and adventitia-cell-exposed VWF [81]. The IVR of FVIII is a lot higher compared to that of FIX, about 2.0–3.0 IU/dL/IU/kg, because the extravascular location of FVIII at the steady state is only 16% of the infused dose against the 40% of FIX [82]. Recently, new information has been achieved about the biology of FVIII [83], even though it is limited to animal models. We now know that the endothelial cell STAB2 receptors in mice allow the binding and the uptake of the FVIII–VWF complex, increasing its clearance and immunogenicity [84,85]. Apart from the impact on the immune system, FVIII shows several extra hemostatic functions which should be taken into account in the treatment of hemophilia A; namely, 1- FVIII and FIX are involved in the maintenance of bone mass and strength [86], vessel physiology, and wound healing [87], and 2- rFVIII infusion may affect the interaction of monocytes with endothelial cells, increasing their migration out the endothelial barrier and the vascular permeability [88].

## 8. Conclusions

The physicians attending the consensus meeting paid great attention to replacement therapy in hemophilia A undergoing surgery. The need for a well-trained MDT, the risk of FVIII inhibitor development, the advantages and disadvantages of CI vs. BI, and the thromboembolic complications were extensively discussed. All participants agreed on the relevance of primary prophylaxis to be implemented just after the first hemarthrosis, much more effectively than secondary prophylaxis, to avoid joint damage in young hemophilia patients. The large availability and safety of third generation FVIII concentrates may allow prompt treatment using both on-demand or prophylaxis treatment. Patients with severe, as well as those with mild, hemophilia A may take advantage of twice-weekly prophylaxis to prevent the occurrence of subclinical joint micro bleedings. There is complete agreement about the possibility that increasing the quality of life when the ABR is zero, which is the primary goal of prophylaxis, will be achieved! A regimen of weekly prophylaxis with rFIX EHL may be very effective in about 30% of hemophilia B because of the long half-life of FIX and its extravascular distribution. The prevalence of missense mutations in hemophilia B (55%) probably allows a baseline concentration of FIX in the subendothelial space, limiting the severity of the disease. Arthropathy seems to be less severe in hemophilia B patients, as borne out by their reduced need for joint replacement compared to severe hemophilia A patients. The quite long half-life of infused rFIX SHL, because of backflow of infused FIX from extravascular to plasma compartment, allows for longer postoperative intervals, 12–24 h, between bolus infusions, or a lower continuous infusion rate.

## Figures and Tables

**Table 1:** Main differences between bolus and continuous infusion.

	Bolus Infusion	Continuous Infusion
Administration of the concentrate	Very easy by peripheral veins punctures or CVC, according to the patient’s condition	CVC is always required for the infusion pump
Cost of the devices	None	The pump is expensive, but the cost of the pump can be amortized by its frequent use
Regulatory issues	Approved	CI is an off-label procedure and must be done under the responsibility of the treater
Bacterial contamination of the concentrate	Minimal risk during the reconstitution of the concentrate	The filling of the disposable plastic bag or reservoir of the pump must be done under sterile conditions
Patient’s mobility restrictions	None for walking patients	Limited using portable mini pumps
The workload for nursing staff	May be heavy, according to the programmed bolus infusions	The pump can be charged with the 24 h dose
Stability of the concentrate at room temperature after reconstitution	No concerns	All concentrates are stable for at least 24 h after reconstitution
Risk of postinfusion very high peaks or low troughs	Possible, according to the infusion rate of the concentrate	The predicted and safe level can be continuously maintained
Monitoring of postinfusion concentration	The trough and the peak, at least once a day, before and after infusions during the first 7–10 days after major surgery are required	Only one daily check, at any time, of the level of replaced concentrate, is enough
The total cost of replacement therapy	Generally, quite high	About 15–30% of the cost can be saved by CI regarding rFVIII SHL or rFVIIa

## Data Availability

The data reported in the paper are not original. The paper is a review of previously published data.
